# Lemierre's Syndrome Associated with Mechanical Ventilation and Profound Deafness

**DOI:** 10.1155/2017/4261429

**Published:** 2017-02-26

**Authors:** Lukas Birkner

**Affiliations:** Department of Internal Medicine, Ev. Krankenhaus Witten gGmbH, University of Witten/Herdecke, Pferdebachstr 27, 58455 Witten, Germany

## Abstract

Lemierre's syndrome is a rare disorder that is characterized by anaerobic organisms inducing a thrombophlebitis of the internal jugular vein (IJV) following a course of oropharyngeal infection. It often occurs in young and healthy patients. Clinicians continuously misinterpret early symptoms until infection disseminates systematically and life-threatening sepsis transpires. We report the case of a 58-year-old female developing Lemierre's syndrome accompanied by invasive ventilation support and a profound deafness requiring the implementation of a cochlear implant. This is one of two reported cases of Lemierre's syndrome associated with mechanical ventilation support and the only case associated with a cochlear implant.

## 1. Introduction

Lemierre's syndrome is a condition caused by primarily anaerobic organisms that induce thrombophlebitis of the internal jugular vein (IJV) and bacteraemia, following a course of oropharyngeal infection [1]. Firstly described in 1936, its incidence and mortality rate decreased drastically after the introduction of antibiotics [2, 3]. Although it is often called “forgotten disease” its occurrence increases since the 1990s [4]. Still Lemierre's syndrome is a rare disease with 0.6–2.3 cases per 1000000 population and a mortality rate of 4–18%. Clinicians frequently misinterpret early symptoms until infection disseminates systematically and life-threatening sepsis transpires [1]. Often the disease occurs in young, healthy patients that show prolonged symptoms of pharyngitis later accompanied by symptoms of septicaemia and pneumonia. Identification of IJV thrombophlebitis as well as cultivation of anaerobic bacteria, mostly* Fusobacterium necrophorum*, confirms the diagnosis [5].

## 2. Case Report 

We report a 58-year-old female patient with a previously fractured elbow. Pain continued throughout the physiotherapy. Swelling of the left arm prolonged over the course of three weeks. Additionally, the patient developed a sore throat, shivering attacks, and fever accompanied by growing nausea. Thereafter she was admitted to the hospital due to a poor general condition, increasing fever, and a developing pharyngitis. On general examination patient was lethargic and disoriented with over 39-degree fever. The patients respiratory condition worsened considerably causing multiorgan failure and a resulting severe pneumonia transpired. Thus, invasive ventilation and administration of catecholamines were started. Ventilator-associated pneumonia could be excluded, because of the sequence of events.

**Figure 1:**
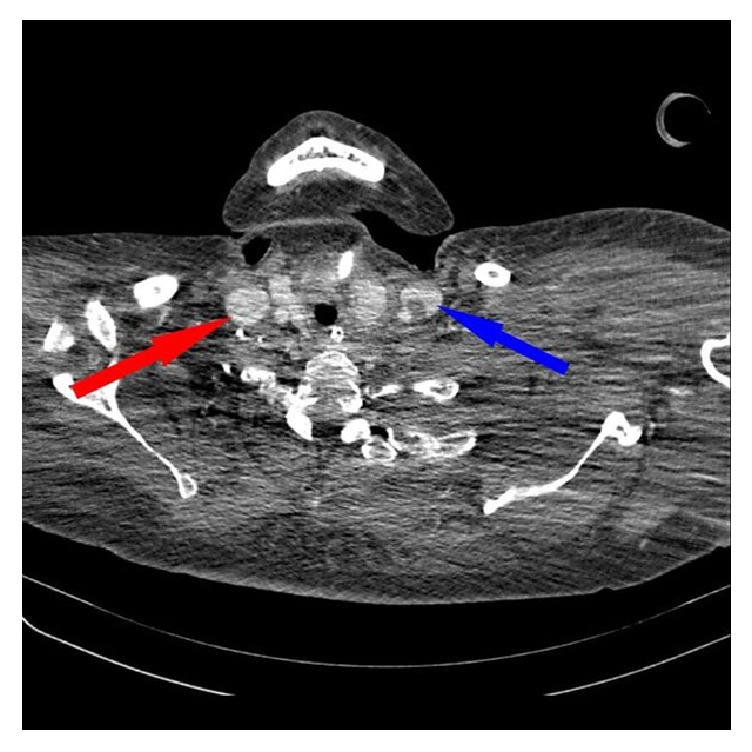
CT scan (axial image) of the thorax with a contrast enhancing agent. Results with thrombus adhering to the wall in both internal jugular veins (left IJV, blue arrow, and right IJV, red arrow).

**Figure 2:**
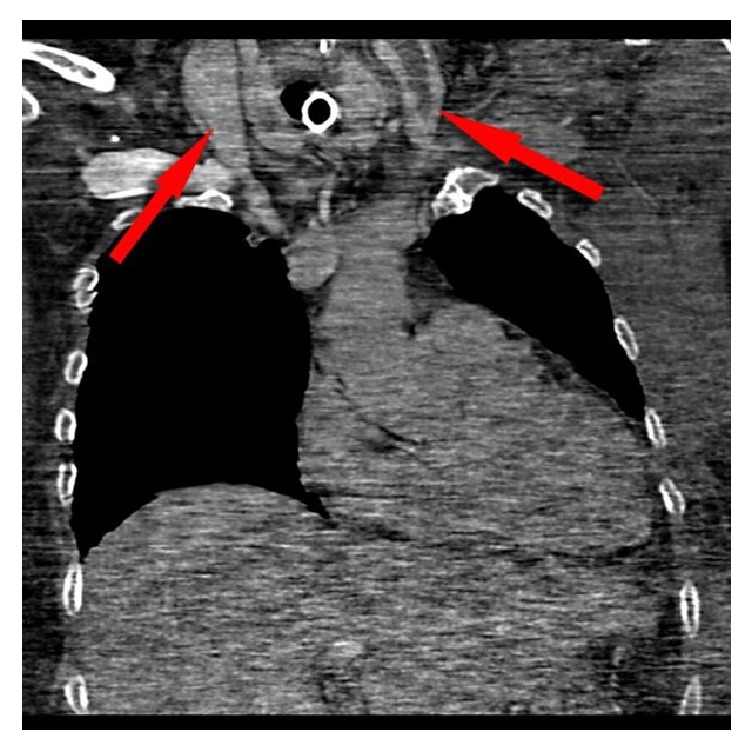
CT scan (coronal image) of the thorax with thrombus adhering to the wall in both internal jugular veins (red arrow).

**Figure 3:**
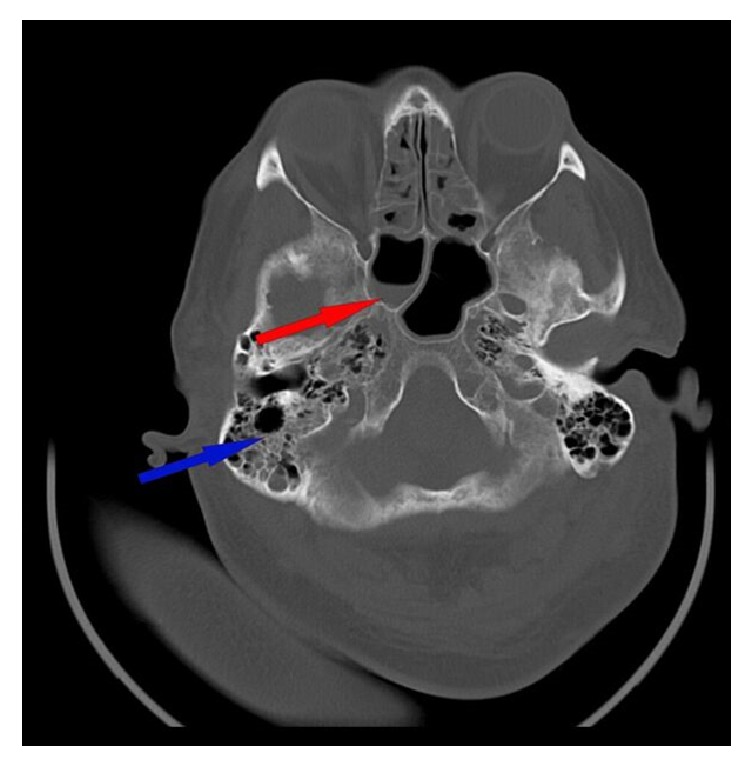
MR imaging of the cranium with bilateral mastoiditis (blue arrow) and sphenoidal sinus with air-fluid level (red arrow). In addition, polypoid swelling of mucous in both ethmoid sinuses.

**Figure 4:**
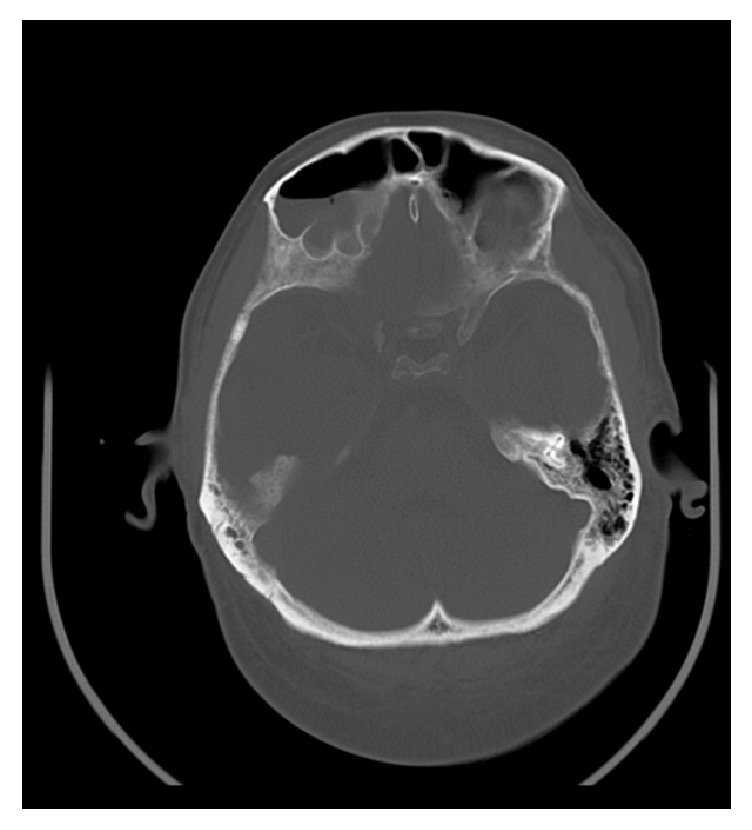
MR imaging of the cranium with air-fluid level in both frontal sinuses.

**Figure 5:**
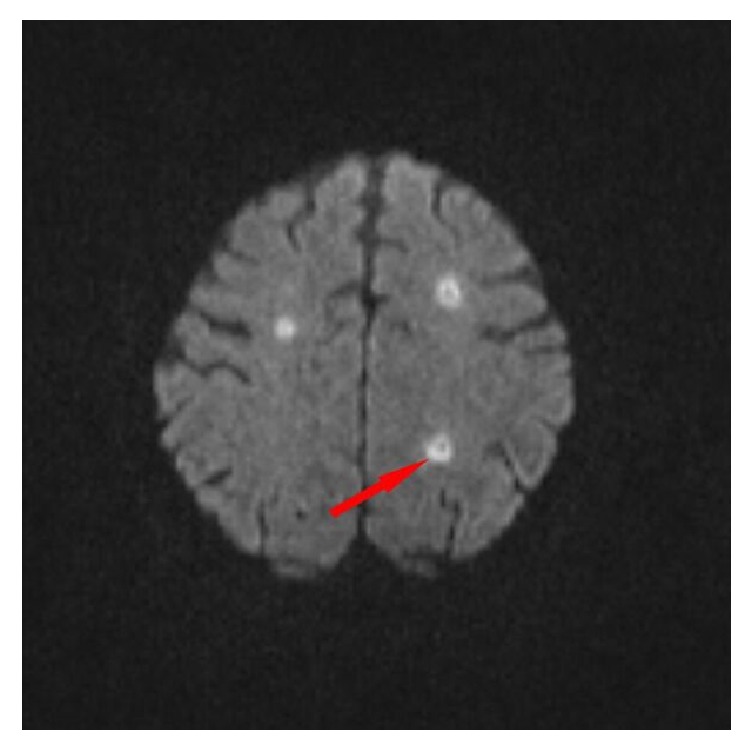
MR imaging of the cranium with multiple small rotund lesions in the sense of septic emboli (red arrow).

Subsequently computed tomography (CT) revealed thrombosis of right and left internal jugular vein (Figures 1 and 2). Furthermore, a bilateral mastoiditis and a chronic sinusitis were discovered (Figures 3 and 4). It must be noted that this could be a coincidental radiological finding. The following magnetic resonance tomography (MRT) displayed multiple sources of infection, likely to be septic emboli, located in the brain (Figure 5). Blood tests revealed a positive anaerobic blood culture bottle. To identify a possible bacterial infection the blood was subcultured. Susceptible testing recognized* Fusobacterium necrophorum *as the responsible agent. The organism was susceptible to penicillin. Antibiotic therapy was reorganized and the patient received metronidazole and penicillin. Based on those findings Lemierre's syndrome was diagnosed. In time the patient's general condition improved and invasive ventilation was stopped. A temporary tracheostomy was performed. Severe dysphagia was discovered and the patient required a PEG (percutaneous endoscopic gastrostomy) feeding tube. Additionally, a profound deafness, which presumably started during the septic shock, complicated communication with the patient. As a result, a cochlear implant was agreed upon and the patient was transferred to a specialized hospital. Intensive physiotherapy enabled the patient to walk for very short distances again.

## 3. Discussion

Lemierre's syndrome is a thrombophlebitis of the IJV originated from an initial oropharyngeal infection. It most commonly occurs in the 2nd decade of life (51%), followed by the 3rd decade (20%), and is least common in the 1st decade (8%) [6]. Clinically the disease presents not unlike pharyngitis and pneumonia. The most common symptom is a sore throat as in our patient. It typically outdates other symptoms by 4-5 days although the time period may extend in some cases. Other symptoms frequently associated with Lemierre's syndrome are neck mass and pain often mistaken for enlarged lymph nodes, ear pain, dental pain, pleuritic chest pain often indicating metastatic infection, dyspnea, hemoptysis, bone and joint pain, and abdominal pain [7]. Discovering the thrombus within the IJV is the first crucial step towards diagnosing Lemierre's syndrome. For visualisation of the IJV ultrasonography, contrast enhanced CT or less frequently MR imagining is used. As cheapest, but most inexact, ultrasonography may miss thrombi with a low echogenicity [8]. Since CT is more effective it is often used for a specific diagnosis as in our case [9]. The second step in diagnosing Lemierre's syndrome is the growth of characteristic anaerobic bacteria from blood culture [7]. In conclusion Karkos et al. suggest that the concurrent presentation of a recent history of oropharyngeal infection, pharyngitis in our case, clinical or radiological evidence of IJV thrombosis, and the isolation of anaerobic pathogens, mainly* Fusobacterium necrophorum,* pose the main diagnostic criteria for Lemierre's syndrome [6]. Although most cases have been associated with* Fusobacterium necrophorum* there have been reports of other cases associated with other bacteria of the genus* Fusobacterium* [10]. Several antibiotics have been proven to be effective against* Fusobacterium*, such as penicillin, lincomycin, clindamycin, and carbenicillin [11, 12]. Of interest, there have been a few strains of* F. necrophorum* that have reported resistance to penicillin due to beta-lactamase production [13].

Mechanical ventilation support has been reported only in one other case associated with Lemierre's syndrome [14]. The sequence of events suggests that the severe respiratory exhaustion and resulting multiorgan failure the patient displayed may be a consequence of the transpiring sepsis most likely due to the* Fusobacterium.* Sepsis is a common condition accompanying Lemierre's syndrome [2, 6]. Furthermore, Lemierre's syndrome has never been accompanied by profound deafness. This could be a rare effect of the disease possibly caused by damage to auditory pathways due to the septic emboli in the brain or by expansion of the mastoiditis resulting in damage of the inner ear. Mastoiditis has been associated with Lemierre's syndrome before, but our case illustrates that it poses a severe side effect clinicians need to be aware of [6].

## 4. Conclusion

Generally, this case proves that the clinical presentation of Lemierre's syndrome can be diverse. Clinicians need to be aware of severe and possibly lethal side effects Lemierre's syndrome can have and be able to distinguish and interpret the few telltale signs correctly. Ongoing oropharyngeal infections should be investigated at once to prevent critical progression of the disease. Possibly lethal complications such as sepsis and multiorgan failure should not be neglected.
